# Understanding Ethnic Inequalities in Cancer Diagnosis in UK Primary Care: A Narrative Review

**DOI:** 10.3390/cancers18203300

**Published:** 2026-10-13

**Authors:** Liz Down, Melissa Barlow, Sarah E. R. Bailey, Auguster Irabor, Callum Webb, Luke T. A. Mounce, Samuel W. D. Merriel, Jessica Watson, Ge Chen, Lucy Kirkland, Tanimola Martins

**Affiliations:** 1 Exeter Collaboration for Academic Primary Care (APEx), University of Exeter, Exeter EX4 4SB, UK; m.barlow@exeter.ac.uk (M.B.); s.e.r.bailey@exeter.ac.uk (S.E.R.B.); l.t.a.mounce@exeter.ac.uk (L.T.A.M.); g.chen2@exeter.ac.uk (G.C.); l.r.kirkland@exeter.ac.uk (L.K.); tom207@exeter.ac.uk (T.M.); 2 Genetics of Complex Traits (GOCT) Group, Department of Clinical and Biomedical Sciences, University of Exeter, Exeter EX4 4SB, UK; cw1245@exeter.ac.uk; 3 Centre for Primary Care and Health Services Research, University of Manchester, Manchester M13 9QQ, UK; samuel.merriel@manchester.ac.uk; 4 Centre for Academic Primary Care (CAPC), University of Bristol, Bristol BS8 2PS, UK; jessica.watson@bristol.ac.uk

**Keywords:** ethnicity, primary health care, neoplasms, diagnosis, healthcare disparities, intersectional framework

## Abstract

Ethnic inequalities in cancer incidence and outcomes are widespread, although the evidence available for people in the UK is more limited than in some other countries. This narrative review details the current evidence relating to ethnicity and differences in primary care cancer diagnosis in the UK and possible contributing factors. In general, cancer rates are higher for White individuals than for Asian or Black individuals in the UK, but for some cancer sites, the inverse is true. There are also differences in stage at diagnosis by ethnic group for some cancer sites. The routes through which cancer inequalities emerge are complex and difficult to attribute to a single patient characteristic. A variety of social, demographic, genetic and health service factors interact to influence outcomes. There is a wide research base demonstrating that Asian and Black individuals in the UK can experience a range of obstacles when interacting with health care services, including cancer investigations. Future research evidence and health service planning needs to include a diverse range of stakeholders in order to decrease cancer inequalities.

## Simple Summary

There are differences in cancer diagnosis rates and outcomes by ethnic group in the UK. For many cancer types, the rates are highest in White patients, but there are a number of cancer sites where this is not true. This review details the current understanding of cancer and ethnicity, with a UK focus, describing factors that are known to contribute to differences in primary care cancer identification. A powerful patient perspective brings into focus some of these issues. We discuss problems with the evidence available, the complexity of understanding and grouping patient experiences, and improvements in research methods to bridge the current evidence gap. We conclude by providing a clinical viewpoint on how the evidence should affect day-to-day practice, as well as the need to push for change.

## 1. Introduction

Health inequalities have widened in recent years for a number of diseases, including cancer [1]. The movement to understand and address these inequalities has led to a re-examination of many health guidelines, particularly where race or ethnicity was used to guide investigations and treatment planning [2]. Despite a wealth of evidence of differences in health outcomes for individuals from different ethnic groups, the causes of these differences and effective actions to remedy them remain elusive. Healthcare practitioners are likely to be aware of some of these issues, but they seldom have guidance on how to tackle them as part of their day-to-day practice.

The majority of research on this topic has been carried out in the USA, with limited evidence from the UK and other countries. This narrative review aims to summarize the current evidence on differences in the primary care aspects of cancer diagnosis by ethnicity in the UK context. While the evidence for this review comes mainly from UK sources, there will be underlying themes that apply equally to populations in other countries.

## 2. Methods

The structure of this narrative review was determined by the authors’ collective understanding of the types of issues we know to be associated with ethnicity and cancer diagnosis. Informal literature reviews were carried out alongside investigating citations of key publications to ascertain the most appropriate evidence in each topic area. PubMed was used for literature searches, with additional use of Google Chrome AI where references were harder to find. Where multiple references were available, for example, for UK cancer incidence, the most recent publication was selected. Publications relating to UK populations were selected where possible to build a picture of cancer diagnosis and ethnicity in a UK context. Supplementary Table S1 gives further details of the evidence included in this review.

Figures were developed to combine and illustrate previously published data; they were produced using R version 4.5.2 “[Not] Part in a Rumble”.

## 3. Ethnicity: Definition, Classification and Limitations

In the UK, ethnicity is routinely collected by healthcare providers. Ethnicity should be self-defined and is typically influenced by “ancestry, culture, identity, religion, language and physical appearance” [3]. Ethnicity categories in the UK have a two-level structure, as detailed in Table 1 [4]. It is important to acknowledge that there is considerable variation in health outcomes within broad ethnic groups as well as between ethnic groups. Ethnicity is considered a risk marker rather than a risk factor [5], in that ethnicity itself is not the cause of increased susceptibility to certain conditions but is associated with causal factors which may or may not be understood.

Most of the research conducted in the UK and the USA investigating ethnicity and health impacts uses standard government classifications of ethnicity. This is because routine data are most likely to be collected in this format, which allows for access to a wealth of supporting information derived from a variety of data sources. There are several criticisms that could be directed at the UK classification: for example, the lack of detail in the Black African category, or the grouping together of those who consider themselves Asian and Asian British. Additionally, many people will not fit neatly into one of the categories, and for those who do not, any research on ethnicity-related health impacts may be of little benefit.

The concept of genetic ancestry is defined by Rebbeck et al. [6] as “inherited DNA that comprises an individual’s genome inclusive of common and rare variations that can be linked to populations with common geographic origins”. This concept is often used in the analysis of genomic data and is distinct from ethnicity.

## 4. Cancer Incidence and Outcomes by Ethnicity

**Figure 1:**
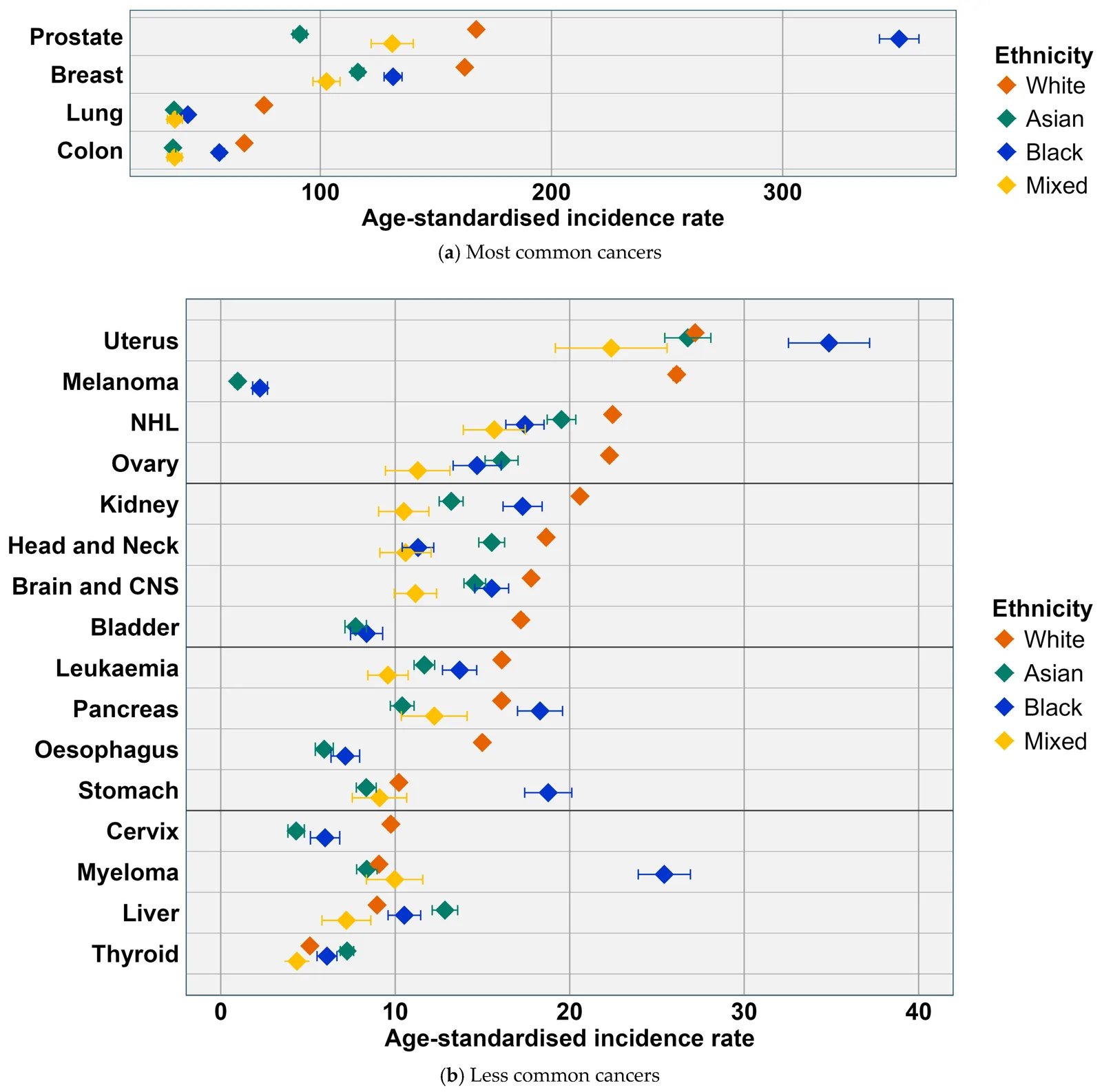
(**a**,**b**) Relative cancer incidence by ethnicity from English cohorts. Error bars show 95% confidence intervals. Age-standardised incidence rates per 100,000 population, 2013–2017, taken from Delon et al. [7]. NHL = Non-Hodgkin Lymphoma, CNS = Central Nervous System. Figures for prostate cancer represent the incidence rate in males, while figures for breast, uterus, ovary and cervical cancer represent the incidence rate in females rather than the whole population. Rates not reported for groups with fewer than 100 cancer cases.

Studies examining cancer incidence and outcomes in the UK have consistently demonstrated significant variation by ethnicity. The incidence of most cancers is highest amongst White individuals and lowest amongst those of Asian ethnicity, but there are notable exceptions [7,8,9]. In comparison to the White population, the Black population has high rates of myeloma, prostate, gallbladder, stomach, and uterine cancer, while the Asian group has higher rates of gallbladder, liver, and thyroid cancer [7] (Figure 1a,b). For individuals of Mixed and Other ethnic groups, incidence patterns are often more variable and less well characterised, partly due to smaller population sizes, with significant heterogeneity within the groups. This makes the interpretation of incidence patterns in these groups very difficult.

**Figure 2:**
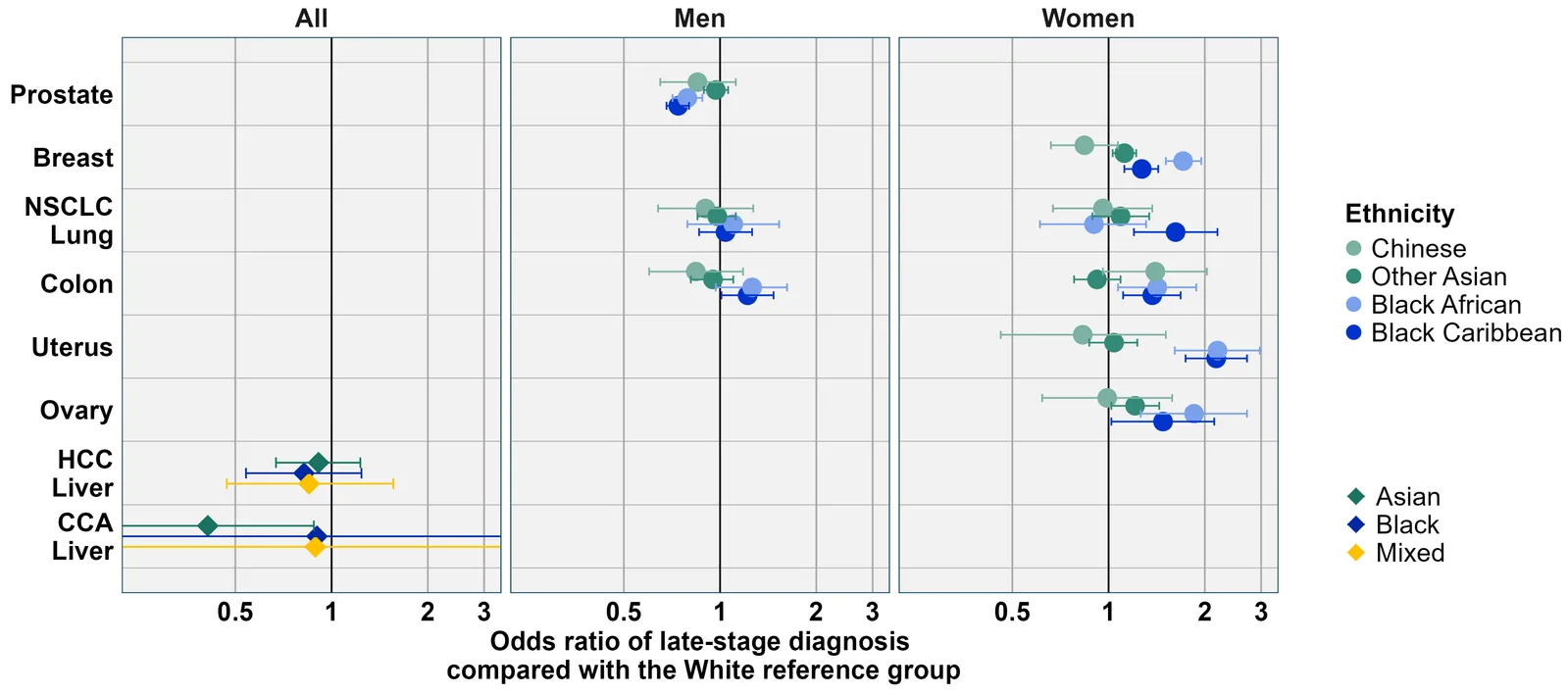
Relative cancer stage at diagnosis by ethnicity from English cohorts. Error bars show 95% confidence intervals. Liver stage data taken from Liao et al. [13]; odds ratio of late-stage diagnosis, 2008–2018, compared to the White reference group, stratified by cancer subtype and adjusted for age, sex, deprivation, and route to diagnosis. Other stage data taken from Fry et al. [11]; odds ratio of late-stage disease, 2012–2016, compared to the White reference group, adjusted for age, comorbidity, deprivation, year of diagnosis and region. NSCLC = non-small cell lung cancer, HCC = Hepatocellular Carcinoma, CCA = Intrahepatic Cholangiocarcinoma.

Lower diagnosis rates in some ethnic groups may reflect genuine differences in risk but may also be due to barriers to accessing medical help [10]. Such barriers can lead to delayed and more advanced-stage diagnosis. A recent analysis of English cancer registry data showed that Asian women were at increased risk of late-stage diagnosis of breast and ovarian cancer compared to White women [11] (Figure 2). The same study reported that Black patients were more likely to be diagnosed at a late stage for cancers of the breast, ovary, uterus, and colon [11] (Figure 2). Birch et al. [12] also found that individuals with colorectal cancer in the Black, Mixed and Other ethnic groups were more likely to be diagnosed with metastatic disease than those in the White group. For less common cancers, the evidence on ethnic variation by diagnostic stage in the UK is limited.

## 5. International Comparisons

While there can be difficulties comparing studies carried out in different populations, especially if healthcare systems are very different, this is a valuable tool to both increase our understanding of the underlying factors that may drive any effects seen and also to improve our insight into how well results will apply in other countries or settings. One recent example of this approach, where routine data was used to assess the role of platelet count in primary care cancer diagnostics, was carried out in England, Canada and Australia, allowing for the validation of these results in a wider variety of settings [14]. Differences in platelet counts between ethnic groups also illustrate the value of considering evidence across different populations: in UK studies, Black individuals tend to have lower platelet counts than White individuals, whereas studies from the USA have generally reported higher platelet counts among Black individuals compared to White individuals [15].

International comparisons are made difficult when ethnicity is involved, as each country may have different definitions of ethnic groups, resulting from historical and current patterns of immigration and changes in terminology over time. The associated demographic features of each ethnic group may also be characterised differently, depending on population dynamics in each area. Differences in healthcare infrastructure and delivery may affect how different demographics interact with and access healthcare, potentially contributing to differences observed between populations There are also a number of countries where the collection of data on race or ethnicity is restricted, particularly in Europe.

## 6. Causes of Differences Between Ethnic Groups

As a result of the complex multifactorial nature of ethnicity, there are many potential avenues by which ethnic differences in cancer outcomes might arise. At the individual level, it is challenging to disentangle the effects of genetic and epigenetic ancestry, cultural and societal factors, and behavioural factors. However, population-level evidence is available, which begins to give an insight into some of the processes that may drive the differences we see in cancer outcomes.

A range of cancer-linked genetic variants have been found to be more common in individuals with non-European ancestry, although the lack of population diversity in large genomic datasets means that individuals of non-European ancestry have been under-represented in genomic research [16]. Developments in epigenetics have identified cancer-linked methylation differences between different ethnic groups, but, as with many of the features we discuss, it is difficult to distinguish the effects of race or ethnicity, deprivation, and environmental conditions [17].

In many countries, there is a strong relationship between ethnicity and economic deprivation. For example, when examining the most deprived half of local areas in the 2021 English census, 47% of White residents lived in these areas, compared to 68% of the Asian population and 78% of the Black population of England [18,19].

The populations of more deprived areas typically have a higher cancer incidence rate, higher cancer mortality, and poorer cancer survival [20]. Studies investigating ethnicity and deprivation alongside cancer outcomes present a complex picture [21,22], making it difficult to be clear as to what extent poor prognosis can genuinely be linked to ethnicity, rather than unmeasured elements of deprivation. There are a number of barriers to healthcare for those living in the most deprived areas, for example higher rates of emergency hospital admissions [23] and longer elective waiting lists [24].

There is evidence that the experience of racism can have further health impacts [25,26], and that an awareness of discrimination can lead to mistrust in healthcare providers [27]. Additionally, patients who are not White report poorer doctor–patient communication [28,29], and the lack of services to overcome language barriers can contribute to inequity in primary care [30]. Cancer fear, stigma, and fatalism are known to decrease help-seeking for non-White patients with potential cancer symptoms [31,32].

## 7. Differences in Primary Care Access and Presentation

It is known that there are often differences between patients of different ethnic groups in terms of cancer symptom awareness [33,34], barriers to seeking medical help [30,33,34], and symptom reporting [35], although the reasons for that variation are likely to be complex, multi-factorial, and heterogenous even among individuals in the same group or category. There is evidence that multiple demographic factors, including recorded ethnicity, impact upon GP consultation rates [36], doctor–patient communication [28], and the resulting route to cancer diagnosis [37]. Recent research has found that the diagnostic interval (time from first reported symptoms to cancer diagnosis) is longer for Black and Asian patients than for White patients in the UK [38].

For those cancers where screening programmes are available, screening uptake is typically lower for patients living in more deprived areas and for those from non-White backgrounds [32,39].

For some blood tests, such as haemoglobin and PSA, differences in typical test values between individuals of different ethnic groups have been observed in multiple studies. When scrutinised further, differences are apparent in many blood test result distributions [15]; possible contributary elements include underlying genetic factors, lifestyle and environmental factors, or chronic health conditions (all four may have an impact).

Given the variety of differences in primary care presentation and assessment when considering demographic factors, it is difficult to know how this is likely to impact patients’ cancer-related outcomes. In particular, for an individual, their ethnicity is experienced in the context of other demographic and health-related factors, which will have an impact on their health and experience of healthcare.

## 8. Precision Medicine and Artificial Intelligence

Genetic susceptibility to individual health conditions can influence the risk of those conditions developing, particularly cancer. The NHS 10-year Plan and the National Cancer Plan project an expansion of genomic testing in the NHS to personalise disease risk assessment and treatment [40,41]. In theory, Genetic Risk Scores (GRS) for cancer could be used in primary care to prioritise further testing for patients who score highly and may enhance risk assessment for current screening programmes for certain cancers. GRS have been developed for prostate [42], breast [43], and several other cancers, including lung and thyroid [44]. NICE (the National Institute for Health and Care Excellence) has also endorsed the GRS-based Breast and Ovarian Analysis of Disease Incidence and Carrier Estimation Algorithm (BOADICEA) model [45,46,47], implying that GRS implementation in the clinic may be feasible in the near future.

However, many GRS have been developed using data from individuals of European ancestry, either exclusively or as a majority of the population [42,43,44]. There is evidence that many GRS, namely those developed for prostate cancer prediction, identify high-risk individuals more effectively in test populations of European ancestry compared with those of non-European ancestry [48,49]. This highlights the need for more ancestry-inclusive models that better predict disease risk across all groups. Without addressing these issues, there is a risk that the expansion of the use of genetic data in cancer risk assessment widens existing inequalities.

The National Cancer Plan also gives emphasis to future development of personalised cancer risk profiling to improve early diagnosis, as well as the use of artificial intelligence (AI) to improve diagnosis and treatment of cancer [41]. These emerging technologies will need to be adopted carefully, as there are many examples of the potential of algorithms to increase inequalities [50,51].

## 9. Problems with the Evidence

The relative role of demographic factors and their potential interactions is not well understood and therefore presents a challenge when interpreting any research relating to ethnicity. For example, the standard measure for deprivation in England is the Index of Multiple Deprivation (IMD), which does not include ethnicity in its calculation. A potential alternative is the Ethnic Group Deprivation Index (EGDI), which allows for further exploration of the interaction between ethnicity and relative deprivation levels [52].

There is a tension in carrying out research that considers ethnicity as a factor, as great care is needed to interpret any associations with ethnicity to understand any causal processes and to ensure that health and other inequalities are not increased by this process. Historically, race- and ethnicity-adjusted medical practices have been applied, with the potential to exacerbate inequalities and increase mistrust in healthcare [2,53]. Despite these concerns, it is clear that there are wide ethnic inequalities in certain health measures in the UK [7,54,55,56], and in the absence of a better way of handling race and ethnicity and investigating these issues, little progress will be made to improve the situation without further research.

The historical under-representation of non-White patients in clinical trials and biobank resources [49,57] represents a real challenge when interpreting the existing evidence. A lack of diversity in research groups is also an issue, which can lead to bias in how research is carried out and interpreted. The involvement of a diverse research community informed by new and varied voices of a similarly diverse group of patient and public representatives with relevant lived experience and trust in the researchers can ensure more appropriate research and greater insight into the key questions that need to be answered.

Box 1 contains a viewpoint from our patient and public involvement and engagement (PPIE) group member, illustrating the power of listening to those with lived experience of health inequalities. These are individual insights and are not intended to represent the views or experiences of Nigerian people more broadly, nor of the other ethnic groups discussed in the paper.

Box 1 **Viewpoint—Patient and Public Involvement and Engagement representative.**

Within my family back in Nigeria, there have been two known cases of breast cancer: my mother and my cousin. My mother died of the disease, while my cousin survived. One critical factor distinguishing the outcome was access to healthcare and financial resources. My cousin was financially advantaged because her immediate family were rich. This enabled early diagnosis and timely access to a specialist cancer treatment facility in Nigeria. This comparison stresses the role of socioeconomic status in shaping cancer survival, even within the same familiar and national circumstances.

Furthermore, my observations of a male friend presently undertaking cancer treatment in the UK further demonstrate how structural and economic limitations influence cancer experiences. Notwithstanding his diagnosis, he continues to work intensively instead of prioritising rest and focusing on his treatment. He attributes this to financial constraint, as his immigration status restricts his access to public funds. This example highlights how socioeconomic factors and deprivation may worsen vulnerability among people with serious illnesses, even within high-income healthcare systems like the UK.

I believe that statistics on cancer incidence and outcomes cannot be fully generalized across populations due to cultural and contextual factors. For example, in Nigeria, self-medication is a common practice, and people purchase medications over the counter without consulting trained professionals. People may also rely on informal vendors who lack formal medical training. Some learn as apprentice from others who are also not qualified themselves, resulting in delayed professional diagnosis. Typically, individuals only seek care at established hospitals after prolonged periods of self-medication, which often leads to diagnosis at a later stage of the disease. These patterns emphasize how cultural practices, accessibility of informal healthcare, and delayed presentation can contribute to observed differences in cancer outcomes.

Within the Black community, cancer is often considered a socially taboo illness, and people find it awkward to openly discuss it. Culturally, some people believe cancer is a form of punishment or misfortune. This leads individuals to disguise their diagnosis or attach their symptoms to other illnesses. Disclosure is often done upon trust, with individuals only happy to share their experiences when they trust who they are sharing it with. This dynamic is similar to the historical coverup of social issues in Nigeria such as domestic violence, which was predominantly covered up until public awareness gave space for disclosure and support. Likewise, consistent speaking about cancer within Black communities would likely increase and be embraced. One way this can be achieved is when there is scientific evidence drawn from research studies.

## 10. How Can Research Evidence Be Improved?

Research that generates results representative of the population as a whole is a crucial goal to improve our understanding of health inequalities and to provide routes for improvement. Indeed, to provide evidence that allows for a better understanding of any differential effects between subgroups, representative research populations might not be enough, and over-sampling of some groups might be necessary [58].

Carefully considered PPIE with diverse groups of participants is a starting point for the design and implementation of inclusive research [59]. The reasons for poor diversity in many health research cohorts are complex, including study design factors, recruitment strategies, and mistrust of healthcare professionals [60]. A number of initiatives are now in place to support researchers to improve this picture [61].

Since 2020, clinical algorithms that take race or ethnicity into account have come under substantial scrutiny for their potential to reinforce existing health inequalities [2]. This has prompted change in many areas of healthcare, for example, the removal of ethnicity adjustment when assessing kidney function in England [62]. Current health service plans to introduce personalised or AI-powered treatment decisions carry some risk of introducing unintended bias to decision making and will need careful ongoing scrutiny [40,63].

Using routinely collected data, health researchers can identify where ethnic inequalities exist and explore their drivers while acknowledging that ethnicity is an imperfect proxy for social and structural factors. Quantitative analysis provides robust evidence of measurable disparities, while mixed-methods insights at community or PPIE level can give context to the patterns.

## 11. Next Steps for Clinicians

For clinicians working in General Practice, it may seem that there is a great deal of emphasis on reducing health inequalities, but it is often unclear what actions can actually be taken. A variety of training resources are available, for example, from the Royal College of General Practitioners [64].

Although there are often, on average, differences in blood test results or potential cancer symptoms between patients from different ethnic groups in the UK, there is not enough evidence to support more tailored diagnostic guidelines. Clinicians should remain cautious about attributing test results to ethnicity, as this could inadvertently exacerbate inequalities. An awareness of how a patient’s experience of racism or other discrimination could affect their interaction with healthcare services is a key step towards reducing inequalities.

Finally, clinicians have a role in advocating for system-level change: providing feedback when clinical pathways inadvertently disadvantage particular populations, supporting inclusive and culturally sensitive service design, and contributing to local quality improvement initiatives aimed at reducing unwarranted variation.

## 12. Conclusions

This review has detailed the current evidence relating to routes to ethnic inequalities in cancer diagnosis in primary care in the UK. The most consistent finding is that cancer incidence and stage at diagnosis vary between ethnic groups, but these patterns differ by cancer site. White people have the highest incidence for many cancers, while higher incidence among Asian or Black people is observed for particular cancer sites, including gallbladder, liver, and thyroid cancer among Asian people and myeloma, prostate, gallbladder, stomach, and uterine cancer among Black people. Late-stage diagnosis is more likely for Asian women diagnosed with breast or ovarian cancer compared to White women, while Black people are more likely to be diagnosed at a late stage for cancers of the breast, ovary, uterus, and colon.

The causes of these differences in cancer incidence are likely to be multifactorial, involving genetic, environmental, social, and structural factors. However, the evidence remains limited and heterogenous, with differences in how ethnic groups are defined and variation between healthcare settings. There is currently insufficient evidence to support modifying cancer diagnostic guidelines or clinical care according to ethnicity alone.

Under-representation of non-White ethnic groups in research populations is an increasing concern as AI and other personalised approaches to cancer detection and risk assessment develop: models trained on poorly representative populations could risk widening existing inequalities.

Further research is needed with diverse patient groups to allow us to better understand the causal pathways involved, identify targets for interventions, and ensure that emerging approaches to cancer detection and risk assessment are effective across diverse populations.

## Figures and Tables

**Table 1:** UK ethnicity categories.

	Percent of the Population of England	
**Asian, Asian British or Asian Welsh**	**9.6%**	
Bangladeshi		1.1%
Chinese		0.8%
Indian		3.3%
Pakistani		2.8%
Other Asian		1.7%
**Black, Black British, Black Welsh, Caribbean or African**	**4.2%**	
African		2.6%
Caribbean		1.1%
Other Black		0.5%
**Mixed or Multiple ethnic groups**	**3.0%**	
White and Asian		0.8%
White and Black African		0.4%
White and Black Caribbean		0.9%
Other Mixed or Multiple **ethnic groups**		0.8%
**White**	**81.1%**	
English, Welsh, Scottish, Northern Irish or British		73.5%
Irish		0.9%
Gypsy or Irish Traveller		0.1%
Roma		0.2%
Other White		6.4%
**Other ethnic group**	**2.2%**	
Arab		0.6%
Any other ethnic group		1.6%

2021 English census population statistics [4].

## Data Availability

No original data is presented in this work.

## Supplementary Materials

The following supporting information can be downloaded at: https://www.mdpi.com/article/10.3390/cancers18203300/s1, Table S1. Evidence included in this review.

## Abbreviations

The following abbreviations are used in this manuscript:

NHL	non-Hodgkin lymphoma
CNS	central nervous system
NSCLC	non-small cell lung cancer
HCC	hepatocellular carcinoma
CCA	intrahepatic cholangiocarcinoma
PSA	prostate specific antigen
GRS	genetic risk score
NICE	National Institute for Health and Care Excellence
AI	artificial intelligence
IMD	Index of Multiple Deprivation
EGDI	Ethnic Group Deprivation Index
PPIE	patient and public involvement and engagement
